# A comparative study of intra canal stress pattern in endodontically treated teeth with average sized canal diameter and reinforced wide canals with three different post systems using finite element analysis

**DOI:** 10.4103/0972-0707.62639

**Published:** 2010

**Authors:** Amandeep Kaur, Meena N, Shubhashini N, Anitha Kumari, Ashish Shetty

**Affiliations:** Department of Conservative Dentistry and Endodontics, V.S. Dental College and Hospital, Bangalore, India

**Keywords:** Cast post and core, carbon fiber post, FEA, FEM, stainless steel post

## Abstract

**Study methodology:**

This is a comparative study of intra canal stress patterns in endodontically treated maxillary central incisor with: average sized canal diameter and wide canals reinforced with three different post systems - cast post and core, carbon fiber post, stainless steel post; restored with ceramic crown using finite element analysis (FEA). All the models were subjected to a force of 100N applied at 450 to the long axis of the tooth at the middle third of the palatal surface of the restored ceramic crown.

**Results:**

The FEA revealed that all the post systems showed maximum stress in the coronal and middle third of the root. Maximum stress was seen on the inner dentinal wall in case of stainless steel post followed by cast gold and carbon fiber post, both in the models without reinforcement as well as in the reinforced models.

## INTRODUCTION

The placement of a dowel – core is a recognized treatment modality to retain final restoration in an endodontically-treated tooth in which a large amount of coronal structure has been lost. The structural rehabilitation of pulpless tooth is an important step to ensure successful function of the tooth in the oral cavity following endodontic treatment.[1,2] This has resulted in the clinically convenient post – core system to help restore lost tooth structures. Posts of various types are available: precious metal cast post or prefabricated posts made of up of stainless steel, titanium, ceramic, carbon fiber, composipost, fiber composite laminate post etc. Cast post and core casting demands extra clinical and laboratory time. The prefabricated posts permit fast, economical and easy clinical placement, but often the shape of the canal has to be modified to match the shape of the post. However, considerable controversy exists regarding the optimal choice of material that can be used for dowel and core in different clinical situations.

It has been established that resistance to fracture is directly related to the amount of remaining tooth structure. Structurally weakened root compromises the prognosis of a long term successful restoration of the tooth. Therefore, in teeth with loss of significant amount of coronal and radicular tooth structure, there is a need to assess alternatives to cast post rehabilitation; a cast post can act as a wedge and increase the chance of root fracture. Many studies suggest the use of dentin-bonded resin composite for intra-radicular reinforcement to help strengthen damaged and weakened root, allowing them to be rehabilitated.

Preferences of dentists have evolved from vey rigid material for post system to a material that closely resembles the properties of dentin, so as to produce a homogeneous unit and result in reduction of stresses in the root structure. Studies suggest the use of dentin bonded resin composite for intraradicular reinforcement to help strengthen damaged and weakened root.[1] There is a strong co-relation between the elastic modulus of the posts and the resulting stress and root fractures.[3,4] Materials such as: carbon fiber post[5,6] have modulus of elasticity, which is nearly identical to that of dentine and reported to cause less stress in the tooth and fewer root fractures.[7]

This study aims to find out the stress generated and hence the suitability of cast post and core, stainless steel post and carbon fiber post in an average sized canal and reinforced wide canal in a maxillary central incisor tooth using finite element model (FEM)[8] analysis.

## MATERIALS AND METHODS

**Modeling:** The first step in FEM analysis is modeling. The quality of analysis depends on the accuracy of the model. The tooth was subjected to C.T. scan and a cross section of the tooth was obtained at an equal interval of 0.5 mm, in DICOM format. Using the software MIMICS these scanned cross sections were converted into a three-dimensional model. Thus a virtual model of maxillary central incisor was obtained. A bone was created as a rectangular block. The root portion was embedded in the bone. This bone was constrained of motion in all directions. The model was exported to ANSYS 10.0 version. The effect of PDL was not taken into consideration.

**Meshing:** The creation of FEM is the next step. The model was subdivided into simpler geometric shapes or elements whose apexes meet to form nodes. This is known ass meshing of the model and was preformed with the computer program – HyperMesh 7.0. Such elements and their nodes remain in contact irrespective of the size and nature of the stresses generated in computer simulation.

The virtual models used were:

- Cast gold post and core, carbon fiber post, stainless steel post with diameter 1/3rd the diameter of the root at the post and root canal filing junction
- Cast gold post and core, carbon fiber post, stainless steel post with diameter 1/3rd the diameter of the root at the post and root canal filing junction and 1mm of reinforcement of the internal surface of the canal with flowable composites

It was assumed that Young's modulus of ligament and cement closely matches that of dentine. The cast – post was modeled by considering the morphological characteristics of the tooth. 5mm of root canal filling of gutta-percha remained at the apical third in all models. Porcelain crown thickness was taken as 1mm.

### Model groups

Model I Cast gold post and core with diameter 1/3rd the diameter of the root at the post and root canal filling junction.

**Table:** 

Model II	Carbon fiber posts with diameter 1/3rd the diameter of the root at the post and root canal filling junction, with composite core of 4 mm.
Model III	Stainless post with diameter 1/3rd the diameter of the root at the post and root canal filling junction, with composite core of 4 mm.
Model IV	Cast gold post and core with diameter 1/3rd the diameter of the root at the post and root canal filling junction after 1 mm of reinforcement of the internal surface of the canal with flowable composites.
Model V	Carbon fiber post with diameter 1/3rd the diameter of the root at the post and root canal filling junction after 1mm of reinforcement of the internal surface of the canal with flowable composites. The model had composite core.
Model VI	Stainless post with diameter 1/3rd the diameter of the root at the post and root canal filling junction after 1mm of reinforcement of the internal surface of the canal with flowable composites. The model had composite core[Table 1]

**Table 1:** Properties of various materials

Material	Modulus of elasticity (GPA)	Poisson's ratio
Dentine	18.6	0.31
Bone	1.37	0.30
Gutta-percha	0.00069	0.45
Cast gold	77	0.33
Carbon fiber	21	0.31
Stainless steel	200	0.33
Composite resin	8.3	0.28
Flowable composite	6.2	0.3
Porcelain	69	0.28

Models were then subjected to a load of 100N at 450 to the long axis of the tooth on the palatal surface at the middle third of the crown and the stress analysis was carried out using the software ANSYS 10.0.

## RESULTS

- All the posts showed similar patterns on the tooth-root surface. Maximum stress was noted on the middle third and coronal third of the root in the range of 11.05-15.735 Mpa.
- Maximum stress on the inner dentinal wall in the models without reinforcement was generated by stainless steel post (39.25-44.11 Mpa), followed by cast gold (15.97-17.87 Mpa) and least by carbon fiber post (4.53-5.04 Mpa) [Table 2].

**Table 2:** Results of model I, II, III

	Surfaces	Labial surface	Lingual surface
Model I [Figure 1]	Outer tooth surface	11.224-12.8 MPa	12.814-14.404 MPa
	Post surface or inner dentine surface	15.974-17.873 MPa	10.278-12.177 MPa
Model II [Figure 2]	Outer tooth surface	11.407-13.023 MPa	13.023-14.639 MPa
	Post surface or inner dentine surface	4.531-5.046 MPa	4.017-4.531 MPa
Model III [Figure 3]	Outer tooth surface	11.052-12.618 MPa	12.618-14.184 MPa
	Post surface or inner dentine surface	39.25-44.117 MPa	29.517-34.382 MPa
- [Table 3] Maximum stress generated in the reinforced model was generated by stainless steel post (7.33-8.21 Mpa), followed by cast gold (7.39-8.28 Mpa) and least by carbon fiber post (2.74-3.04 Mpa). Reinforcement with flowable resin resulted in reduction of stress.

**Table 3:** Results of model IV, V, VI

	Surfaces	Labial surface	Lingual surface
Model IV [Figure 4]	Outer tooth surface	12.11-13.826 MPa	13.826-15.542 MPa
	Inner dentine surface	7.397-8.289 MPa	7.397-8.289 MPa
	Post surface	17.74-19.858 MPa	13.505-15.623 MPa
Model V [Figure 5]	Outer tooth surface	12.261-13.998 MPa	13.998-15.735 MPa
	Inner dentine surface	2.741-3.047 MPa	2.741-3.047 MPa
	Post surface	5.018-5.594 MPa	3.867-4.443 MPa
Model VI [Figure 6]	Outer tooth surface	11.936-13.628 MPa	13.628-15.32 MPa
	Inner dentine surface	2.885-3.77 MPa	7.33-8.219 MPa
	Post surface	43.563-48.977 MPa	38.148-43.563 MPa

**Figure 1:**
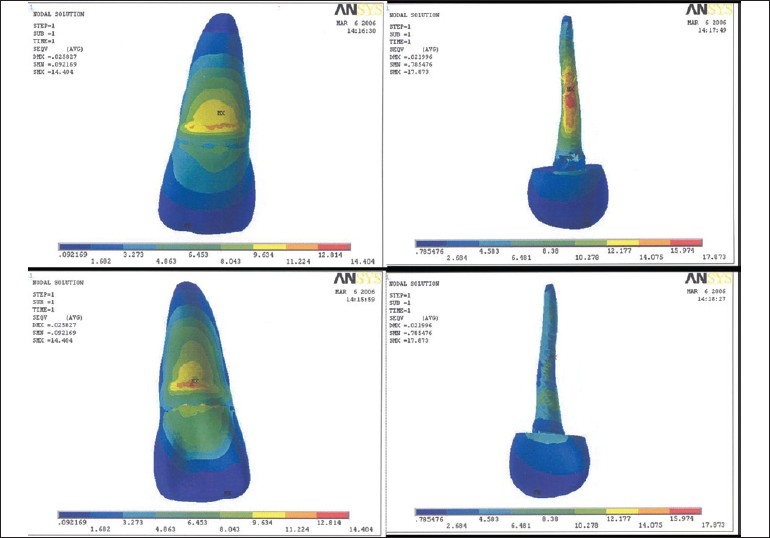
Stress pattern in model I - labial surface, labial surface of post, lingual surface, lingual surface of post

**Figure 2:**
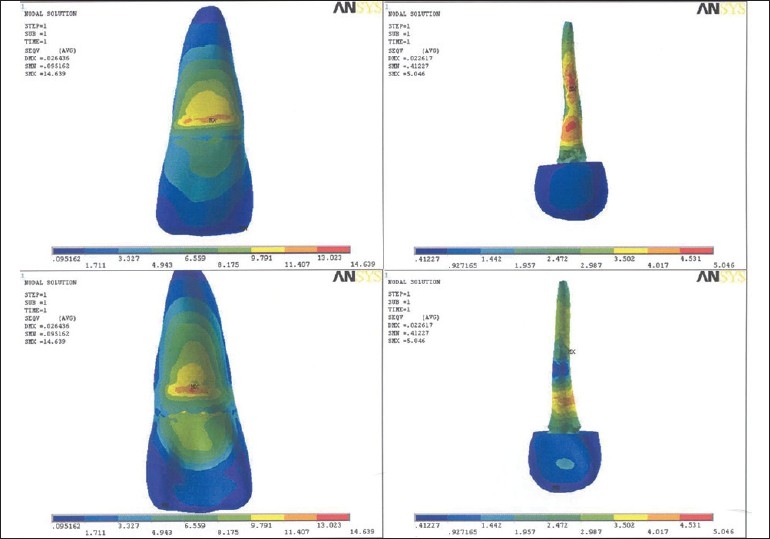
Stress pattern in model II - labial surface, labial surface of post, lingual surface, lingual surface of post

**Figure 3:**
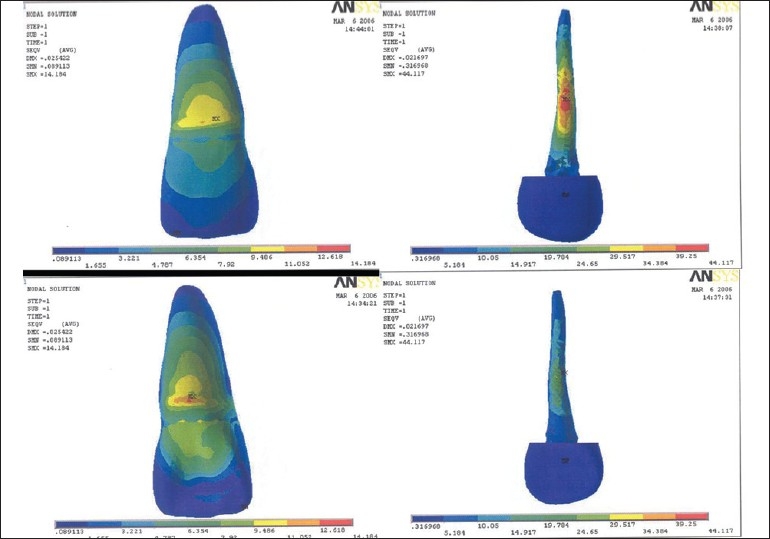
Stress pattern in model III - labial surface, labial surface of post, lingual surface, lingual surface of post

**Figure 4:**
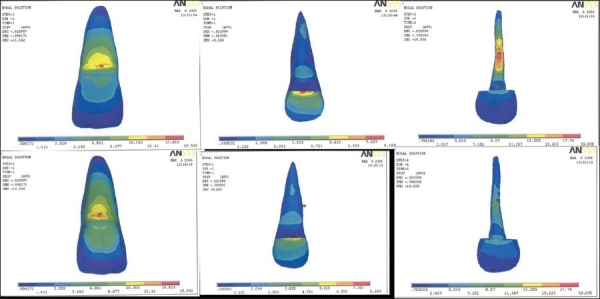
Stress pattern in model IV - labial surface, labial inner dentinal surface, labial surface of post, lingual surface, lingual inner dentinal surface, lingual surface of post

**Figure 5:**
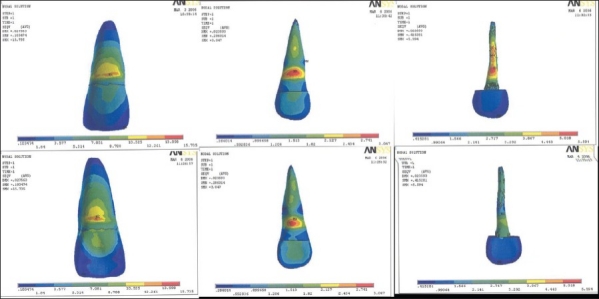
Stress pattern in model V - labial surface, labial inner dentinal surface, labial surface of post, lingual surface, lingual inner dentinal surface, lingual surface of post

**Figure 6:**
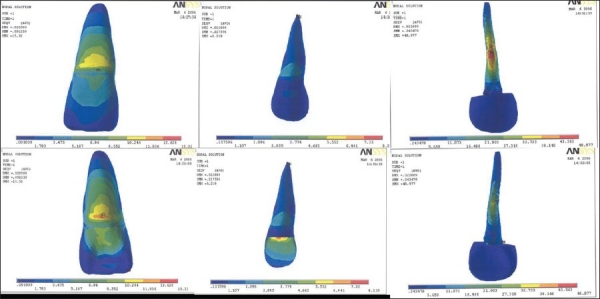
Stress pattern in model VI - labial surface, labial inner dentinal surface, labial surface of post, lingual surface, lingual inner dentinal surface, lingual surface of post

## DISCUSSION

The functional and para-functional forces that occur within the mouth result in extremely complex structural response by the oral tissues. This makes oral rehabilitation an inherently challenging task.[9] The important criteria for post core design include: i) to provide sufficient retention to the core and ii) to distribute functional stresses uniformly through-out the tooth-root.[10] A variety of post systems exist for the restoration of endodontically treated teeth that have inadequate remaining tooth structure. Caputo and Standlee stated “the price for more retention is the increasing risk of damaged tooth structure”. The high success rate for modern day endodontics has resulted in an increased demand for clinically convenient post and core systems to help restore lost tooth structure.[5,11,12]

In 2004, Anil Kishen *et al.* suggested that the structure of inner dentin, which surrounds the root canal is less mineralized and has more collagen, hence posses low modulus of elasticity.[13] The conservation of the inner dentin is crucial to offer toughness or fracture resistance to the tooth structure.[14] Undue loss or removal of inner dentin would compromise the toughness criteria in dentin structure, which in turn would predispose such a tooth to catastrophic fracture.[15]

Flared canals, whether resulting from carious extension, pulpal pathology or endodontic access, present a restorative management problem. Intra-radicular rehabilitation, before post cementation or post fabrication increases the chance for clinical success of the tooth.[16] It is important that the remaining dentin structure has sufficient strength to support the post core-crown complex that will eventually restore the tooth in form and function.[17,18]

Lack of dentin support at the coronal end of the root canal also poses a problem to the restorative dentist. Conventional procedure of metal ore extension into the defective area may cause fracture of the weakened root structures under forces of cementation or mastication.[19] Reinforcement by means of pins becomes difficult due to lack of dentin structure. To restore the lost dentin, in 1987, Lui *et al.* advocated the use of composite resin as a lining of the root canal surface to reinforce the weakened canal walls.[20] Use of resins for the rehabilitation of a root canal is also supported by Saupe *et al.* in 1996. The modulus of elasticity of composite resin approaches that of dentin.[13] The replacement and reinforcement of intra-radicular tooth structure with a material that is elastically compatible with dentin is far better than morphologic dowel, which has higher modulus of elasticity and hence higher potential to transfer and concentrate applied stresses to the surrounding compromised root structure.[21] The rationale for the use of dentin bonded composite for intra-radicular rehabilitation is well established.

In this study, virtual models of cast-post and core, carbon fiber post along with composite core[22] are considered to restore average sized root canals and widened root canals. Widened root canals were reinforced with 1 mm flowable composite resin. All models were restored with porcelain crowns. The objective of this study was to compare the intra-canal stress pattern in an average sized canal and widened root canal after reinforcement, when restored by the above mentioned post systems. The study aims to throw light on safe utilization of a post material in a root canal with considerations only for the mechanical aspect, as it is an *in vitro* virtual model study. The study indicates that in all the models the stress pattern observed on the outer surface of the tooth was similar. They varied only in value. Maximum stress was observed in the middle third or the coronal third of the outer surface of the tooth. This was in accordance with the study done by Min Hsun *et al.* in 1995[7]and Yaman and Yaman in 1998.[10]

Maximum tensile stress was seen on the labial / lingual surface ranging from 12.81-14.40 MPa (cast gold), 13.02-14.63 MPa (carbon fiber), 12.61-14.18 MPa (Stainless steel). Stresses on the outer surface were approximately same for all the posts whether the canal was reinforced or not. But when viewed on the internal surface of the canal the Von Mises values varied considerably. In an average sized canal the maximum stress was 17.8 MPa for cast gold, 5 MPa for carbon fiber, 44 MPa for stainless steel. This implies carbon fiber exerts least stresses followed by cast post. Maximum stress was exerted by stainless steel post on the internal surface of an average sized canal. This could be attributed to rigid metal posts, which probably cause stress concentration followed by root fracture. This is in accordance to studies done by Isidor *et al.* in 1996 and Purton and Payne in 1996.[23]

It was also observed that composite reinforcement of the canal resulted in considerable reduction of internal stress. Minimum stress of 3.04 MPa was seen with carbon fiber post where as stainless steel and cast post showed equal stress value of 8.2 MPa. The results can be co-related with the modulus of elasticity of carbon fiber. This is supported by a 1989 study by Assif *et al.* which concludes that rigid post causes more accumulation of stresses. They also stated that the thickness of dentin wall is directly proportional to the ability of the tooth to withstand forces. Giovanii *et al.* state that Carbon fiber post has a Young's modulus approximate that of natural teeth, with resulted in decreased stress concentration and therefore an increased longevity of the restoration.[24] The teeth should be restored with a material with modulus of elasticity that resembles root dentin and does not exert crushing or shear forces. Carbon fiber post and composite resin may meet these requirements.

## CONCLUSION

Stress patterns seen in the tooth-root surface were similar in nature, irrespective of the post material used. Maximum stress was seen in case of stainless steel post followed by cast gold and carbon fiber post. The stress generated on the root surface could be co-related to the Young's modulus of elasticity of the material used for the post system. It can be concluded that in widened root canal reinforcement with suitable material, use of carbon fiber post is better than stainless steel post or cast gold post.
